# Clinical associations and prognostic implications of 6-minute walk test in rheumatoid arthritis

**DOI:** 10.1038/s41598-022-21547-z

**Published:** 2022-11-04

**Authors:** Maria Betânia Ferreira, Francisca A. Saraiva, Tomás Fonseca, Rita Costa, António Marinho, José Carlos Oliveira, Henrique Cyrne Carvalho, Patrícia Rodrigues, João Pedro Ferreira

**Affiliations:** 1 UMIB - Unit for Multidisciplinary Research in Biomedicine, ICBAS - School of Medicine and Biomedical Sciences, University of Porto, Porto, Portugal; 2 Hospital da Luz Arrábida, Praceta de Henrique Moreira 150, 4400-346 Vila Nova de Gaia, Portugal; 3 Department of Surgery and Physiology, Cardiovascular R&D Center-UnIC@RISE, Faculty of Medicine of the University of Porto, Porto, Portugal; 4 Hospital de Santo António (HSA), Centro Hospitalar do Porto (CHUPorto), 4099-001 Porto, Portugal; 5 Centro Hospitalar de Entre o Douro e Vouga, Aveiro, Portugal; 6 Université de Lorraine, INSERM, Centre d’Investigations Cliniques Plurithématique 1433, Inserm U1116, CHRU de Nancy and F-CRIN INI-CRCT, Nancy, France

**Keywords:** Rheumatoid arthritis, Outcomes research

## Abstract

The clinical associations and prognostic implications of the 6-minute walk test (6MWT) distance in patients with rheumatoid arthritis (RA) is yet to be explored. To identify the clinical features and prognostic implications associated with the 6MWT in patients with RA. Cohort study including 387 RA patients who underwent 6MWT. Regression models (linear and logistic) were built to identify independent predictors of shorter 6MWT distance. Cox proportional models were used to study the association of 6MWT distance with cardiovascular outcomes. Patients were subdivided according to 6MWT tertiles: 126 patients walked > 405 m, 129 walked 345-405 m, and 132 walked < 345 m. Older age (> 55 years), elevated waist circumference, NT-pro BNP > 125 pg/mL, anemia, C-reactive protein ≥ 3 mg/dL, and troponin T ≥ 14 pg/mL were independent predictors of walking shorter distances. Patients walking less than 345 m had higher risk of a subsequent cardiovascular hospitalization or cardiovascular death compared with patients walking 345 m or more (adjusted HR: 2.98, 95%CI: 1.37–6.51, p = 0.006). Older age, abdominal obesity, anemia, cardiac dysfunction, and inflammation were associated with walking shorter distances in patients with RA. Walking less than 345 m in the 6MWT was associated with a poor cardiovascular prognosis. The 6MWT is simple, reproducible, and inexpensive, easily performed in routine practice, and provides important information regarding the patients´ status and outcomes, enabling the monitorization of the therapeutic optimization of the various domains of the RA.

## Introduction

The 6-minute walk test (6MWT) is a simple, reproducible, and inexpensive method that allows to assess patients` physical capacity^1,2^. The 6MWT reflects well daily physical capacity, and has shown good correlation with quality of life measures and exercise tolerance (e.g., exercise duration and oxygen uptake at the peak of exercise)^3,4^. Importantly, the distance walked in the 6MWT is strongly associated with prognosis in chronic cardiovascular and respiratory conditions^5–12^.

Data on the clinical correlations and prognostic implications of the 6MWT in patients with rheumatoid arthritis (RA) are scarce. To the best of our knowledge, to date, no study has evaluated the factors independently associated with the 6MWT distance in patients with RA nor the prognostic implications of the distance walked. Being RA a multisystemic disease, affecting not only the osteoarticular, but also the cardiovascular and respiratory systems^13^, the 6MWT may serve as an inexpensive tool to comprehensively assess the disease severity, symptom burden and prognosis among patients with RA.

Using a prospective cohort of patients with RA who have performed 6MWT at baseline, we aim to study the clinical correlates and the prognostic implications of the 6MWT in this population.

## Results

### Baseline characteristics of the patients by tertiles of 6MWT

A total of 387 patients (97% of the overall population of 399 patients) had performed a 6MWT. Among these, 126 (32.6%) walked more than 405 m, 129 (33.3%) walked between 345 and 405 m, and 132 (34.1%) walked less than 345 m. Compared with patients that walked more than 405 m, those that walked less than 345 m were older (median 67 vs. 50 years), had higher body mass index and waist circumference, more frequent dyslipidemia, history of myocardial infarction, angina pectoris, stroke, atrial fibrillation, more frequent echocardiographic alterations, dyspnea, fatigue, anxiety and depression, longer duration of RA (median 10.7 vs. 6.5 years), more frequent DAS28 above 3.2 (33.3 vs. 11.9%), poorer renal function, higher proportion of NT-pro BNP above 125 pg/mL (54.5% vs. 12.7%), more frequent troponin T above 14 pg/mL (19.7% vs. 2.4%), and C-reactive protein above 3 mg/dL (59.8 vs. 40.5%). Prescribed medications reflected the severity of the underlying disease. Compared with patients that walked more than 405 m, those that walked less than 345 m received more corticosteroids, and less ACEi/ARBs and beta-blockers. Patients who walked between 345 and 405 m had characteristics compatible with an “intermediate” risk Table 1.

**Table 1:** Baseline characteristics of the patients by tertiles of 6MWT.

Characteristic	6MWT distance (tertiles)			P-value
	> 405 m	345-405 m	< 345 m	
N. = 387	126	129	132	-
Age, years	50.0 (41.0, 60.0)	60.0 (50.0, 67.0)	67.0 (61.0, 74.0)	< 0.001
**Age categories**				< 0.001
≤ 55 years	82 (65.1%)	42 (32.6%)	18 (13.6%)	
56–65 years	32 (25.4%)	42 (32.6%)	37 (28.0%)	
> 65 years	12 (9.5%)	45 (34.9%)	77 (58.3%)	
Male	37 (29.4%)	22 (17.1%)	30 (22.7%)	0.065
**BMI categories**				< 0.001
< 25 kg/m^2^	65 (51.6%)	49 (38.0%)	45 (34.1%)	
25–30 kg/m^2^	50 (39.7%)	53 (41.1%)	47 (35.6%)	
> 30 kg/m^2^	11 (8.7%)	27 (20.9%)	40 (30.3%)	
WC (elevated)	33 (26.2%)	68 (52.7%)	88 (66.7%)	< 0.001
Diabetes	12 (9.5%)	16 (12.4%)	22 (16.7%)	0.23
Dyslipidemia	41 (32.5%)	61 (47.3%)	80 (60.6%)	< 0.001
MI/Angina/Stroke/AFib	3 (2.4%)	9 (7.0%)	23 (17.4%)	< 0.001
SBP, mmHg	128.0 (114.0, 140.0)	133.0 (122.0, 144.0)	133.5 (123.0, 148.5)	0.011
SBP > 140 mmHg	32 (25.4%)	44 (34.1%)	49 (37.1%)	0.11
DBP, mmHg	75.0 (68.0, 82.0)	76.0 (68.0, 83.0)	77.0 (69.0, 83.5)	0.55
DBP > 90 mmHg	14 (11.1%)	14 (10.9%)	15 (11.4%)	0.99
Heart rate, bpm	78.0 (70.0, 88.0)	79.0 (71.0, 89.0)	78.0 (71.0, 88.5)	0.63
Heart rate > 75 bpm	80 (63.5%)	81 (62.8%)	86 (65.2%)	0.92
LVEF, %	62.5 (57.3, 67.0)	61.0 (56.0, 66.0)	60.0 (55.0, 64.0)	0.040
LVEF < 55%	19 (15.1%)	17 (13.2%)	31 (23.5%)	0.064
Echo. alterations^a^	82 (65.1%)	109 (84.5%)	125 (94.7%)	< 0.001
Dyspnea or fatigue	66 (52.4%)	93 (72.1%)	112 (84.8%)	< 0.001
RA dx years, years	6.5 (3.6, 12.4)	8.1 (4.9, 19.0)	10.7 (5.9, 20.7)	< 0.001
RA > 10 dx years	42 (33.3%)	57 (44.2%)	70 (53.0%)	0.006
Articular erosions	34 (27.0%)	45 (34.9%)	55 (41.7%)	0.046
History of other AID	5.0(2.0, 8.0)	7.0 (3.0, 10.0)	6.0 (3.0, 10.0)	0.056
DAS28 with ESR	2.5 (1.9, 2.8)	2.6 (2.2, 3.1)	2.6 (2.3, 3.6)	< 0.001
DAS28 with ESR > 3.2	21 (16.7%)	29 (22.5%)	44 (33.3%)	0.006
HADS A (points)	5.0 (2.0, 8.0)	7.0 (3.0, 10.0)	6.0 (3.0, 10.0)	0.056
HADS D (points)	3.0 (1.0, 5.0)	4.0 (2.0, 9.0)	6.0 (2.0, 10.0)	< 0.001
Epworth scale	2.0 (0.0, 5.0)	2.0 (0.0, 6.0)	1.0 (0.0, 4.0)	0.068
Hemoglobin, g/dL	13.5 (12.7, 14.5)	13.3 (12.5, 14.1)	12.6 (11.6, 13.9)	< 0.001
Anemia	15 (11.9%)	14 (10.9%)	44 (33.3%)	< 0.001
eGFR, mL/min/1.73 m^2^	98.0 (88.0, 109.0)	88.0 (75.7, 102.0)	85.0 (69.0, 95.5)	< 0.001
eGFR < 60 mL/min	6 (4.8%)	7 (5.4%)	18 (13.6%)	0.013
NT-pro BNP, pg/mL	47.0 (28.5, 87.7)	85.5 (45.8, 165.2)	152.3 (81.3, 295.4)	< 0.001
NT-pro BNP > 125 pg/mL	16 (12.7%)	42 (32.6%)	72 (54.5%)	< 0.001
Troponin T pg/mL	2.0 (2.0, 5.0)	4.0 (2.0, 7.0)	7.0 (5.0, 11.0)	< 0.001
Troponin T ≥ 14 pg/mL	3 (2.4%)	10 (7.8%)	26 (19.7%)	< 0.001
CRP, mg/dL	2.0 (0.9, 4.9)	3.6 (1.2, 8.1)	4.2 (1.7, 11.7)	< 0.001
CRP ≥ 3 mg/dL	51 (40.5%)	71 (55.0%)	79 (59.8%)	0.005
ACEi or ARBs	26 (20.6%)	50 (38.8%)	70 (53.0%)	< 0.001
Beta-blockers	5 (4.0%)	11 (8.5%)	25 (18.9%)	< 0.001
Loop diuretics	0 (0.0%)	4 (3.1%)	6 (4.5%)	0.064
Statins	28 (22.2%)	46 (35.7%)	71 (53.8%)	< 0.001
Corticosteroids	43 (34.1%)	49 (38.0%)	85 (64.4%)	< 0.001
Methotrexate	75 (60.0%)	85 (65.9%)	81 (61.8%)	0.61
NSAIDs	20 (15.9%)	44 (34.1%)	35 (26.5%)	0.004
“Biologicals”	24 (19.0%)	26 (20.2%)	23 (17.4%)	0.85
6MWT total distance, m	450.0 (420.0, 465.0)	375.0 (360.0, 390.0)	296.5 (210.0, 330.0)	< 0.001
Stopped 6MWT before 6 min	0 (0%)	1 (0.8%)	6 (4.5%)	0.013

6MWT, 6-minute walking test; BMI, body mass index; WC, waist circumference ≥ 102 cm in men and ≥ 88 cm in women; MI, myocardial infarction; AFib, atrial fibrillation; SBP, systolic blood pressure; DBP, diastolic blood pressure; LVEF, left ventricular ejection fraction;^a^echocardiographic alterations defined by a left atrial volume indexed > 34 ml/m^2^ or left ventricular hypertrophy or E/e’ > 13 or septal or lateral e’ > 9; RA, rheumatoid arthritis; DAS28 with ESR, disease activity score 28 with erythrocyte sedimentation rate; AID, autoimmune disease; eGFR, estimated glomerular filtration rate; NT-pro BNP, N-terminal pro brain natriuretic peptide; CRP, C-reactive protein; ACEi or ARBs, angiotensin converting enzyme inhibitors or angiotensin receptor blockers; NSAID, non-steroidal anti-inflammatory drugs.

### Factors independently associated with less distance walked in the 6MWT

In multivariable stepwise linear regression models, the factors independently associated with walking less in the 6MWT were: older age (56–65 years and > 65 years vs. < 55 years), elevated waist circumference (≥ 102 cm in men and 88 cm in women), NT-pro BNP > 125 pg/mL, C-reactive protein ≥ 3 mg/dL, troponin T ≥ 14 pg/mL, and anemia (hemoglobin < 13 g/dL in men and < 12 g/dL in women) (p < 0.05 for all) Table 2. Similar factors were independently associated with walking less than 345 m in the 6MWT in multivariable stepwise logistic regression models: older age (56–65 years and > 65 years vs. < 55 years), elevated waist circumference, NT-pro BNP > 125 pg/mL, and anemia (p < 0.05 for all) Table 2.

**Table 2:** Factors independently associated with less distance walked in the 6MWT.

6MWT continuous (per minus 10 m walked)		
Variable	Beta coef. (95%CI)	P-value
Age < 55 years	Ref.	–
Age 56-65 years	3.48 (1.59–5.37)	< 0.001
Age > 65 years	7.84 (5.87–9.81)	< 0.001
WC (elevated)	2.95 (1.41–4.96)	< 0.001
NT-pro BNP > 125 pg/mL	3.23 (1.50–4.96)	< 0.001
CRP ≥ 3 mg/dL	2.24 (0.71–3.75)	0.004
Troponin T ≥ 14 pg/mL	3.01 (0.33–5.68)	0.028
Anemia	2.05 (0.06–4.04)	0.043

6MWT categorical (< 345 m vs. ≥ 345 m)		
Variable	OR (95%CI)	P-value
Age < 55 years	Ref.	–
Age 56-65 years	2.95 (1.49–5.83)	0.002
Age > 65 years	5.74 (2.99–11.0)	< 0.001
Anemia	3.33 (1.81–6.13)	< 0.001
WC (elevated)	2.57 (1.56–4.25)	< 0.001
NT-pro BNP > 125 pg/mL	2.15 (1.29–3.59)	0.003

WC, waist circumference ≥ 102 cm in men and ≥ 88 cm in women; NT-pro BNP, N-terminal pro brain natriuretic peptide; CRP, C-reactive protein; OR, odds ratio.

All variables with a P-value of less than 0.05 in Table 1 were entered a stepwise forward model and Table 2 presents the variables retained in the final model.

The beta coefficient represents an association of the variables per each minus 10 m walked from a linear regression model.

The OR represents an association of the variables with walking less than 345 m in 6MWT from a logistic regression model.

### Association of 6MWT distance and cardiovascular outcomes

**Figure 1:**
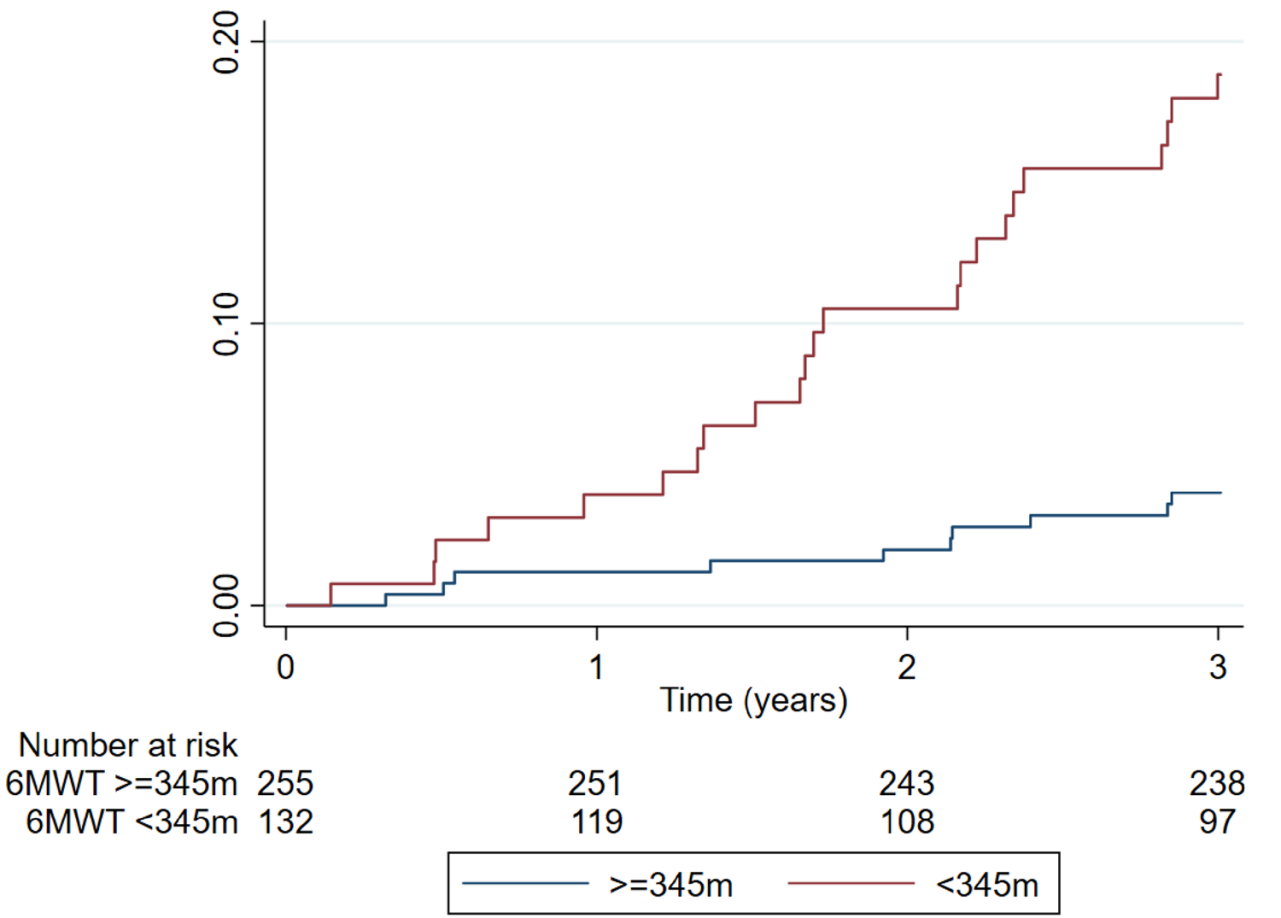
Kaplan–Meier failure curve comparing patients who walked less than 345 m with those who walked 345 m or more in the 6MWT. 6MWT, 6-minute walking test.

Patients walking less than 345 m had the highest risk of cardiovascular events; those walking between 345 and 405 m presented a similar risk to those walking more than 405 m: HR (95%CI) 7.69 (2.69–22.0), p < 0.001 for walking < 345 m, and HR (95%CI) 1.80 (0.53–6.13), p = 0.35 for walking between 345 and 405 m, compared with > 405 m Supplemental Table 1. Patients walking less than 345 m had a three-fold higher risk of a subsequent cardiovascular event (cardiovascular hospitalization or cardiovascular death) compared with patients walking 345 m or more: adjusted HR (95%CI) 2.98 (1.37–6.51), p = 0.006 Table 3 and Fig. 1.

**Table 3:** Association of 6MWT with “hard” cardiovascular outcomes.

6MWT	Walking < 345 m	Walking ≥ 345 m	HR (95%CI)	P-value
	Events, n (%)	Events, n (%)		
“Hard” CV outcome	27 (20.5%)	11 (4.3%)	2.98 (1.37–6.51)^a^	0.006
			2.83 (1.28–6.27)^b^	0.010

6MWT, 6-min walking test; CV, cardiovascular.

“Hard” cardiovascular outcome is a composite of cardiovascular death or hospitalization for cardiovascular causes.

^a^Model adjusted on age, waist circumference, NT-pro BNP, and anemia.

^b^Model adjusted on age, waist circumference, NT-pro BNP, anemia, hypertension, diabetes, angina pectoris, heart failure history, and use of statins.

## Discussion

Our study with RA patients shows that older age, abdominal obesity, elevated NT-pro BNP, c-reactive protein, troponin, and anemia were independently associated with a shorter 6MWT distance. A distance below 345 m was independently associated with poor cardiovascular outcomes. These findings suggest that in RA patients, the 6MWT reflects factors related with ageing, obesity, inflammation, heart failure and anemia, allowing a comprehensive assessment of the patient’ status and prognosis with a simple and inexpensive tool. In routine practice, performing a 6MWT may provide prognostic information and serve as an objective assessment of patients` exercise capacity, allowing a better monitorization of the clinical course of RA and related conditions. The 6MWT is a reproducible method that can be performed in most RA patients (even when exercise capacity in limited by severity of disease or multiple co-morbidities) and may provide reliable information about the patient’s daily activity.

Beyond age, which is an expected factor conditioning walking distance, patients who walked shorter distances, had higher RA activity with higher DAS28 scores and increased levels of c-reactive protein, which is a well-established marker of disease activity and radiologic progression in RA^14,15^. In patients with systemic sclerosis, older age and increased levels of c-reactive protein were also factors independently associated with shorter 6MWT distance^16^.

Anemia was a strong and independent predictor of walking shorter distances in the 6MWT. Anemia is present in 30–60% of RA patients, and it is characteristically of inflammatory origin^17^. Anemia may be a predictor of joint disease activity (RA patients with anemia are more likely to have severe joint disease) and structural damage^18^. Treating anemia may improve the functional capacity of RA patients^17^.

Another important comorbidity independently associated with less distance walked in the 6MWT was visceral obesity (higher waist circumference). Abdominal obesity is an independent risk marker of cardiovascular morbidity and mortality^19^. In RA, obesity is associated with elevated inflammatory markers, chronic pain and disability; which may explain the association between abdominal obesity with shorter distances walked as found in our study^19^.

As we previously reported, our RA cohort had a considerably high prevalence of heart failure (32%), which was previously under-diagnosed^20^. In this regard, performing a 6MWT can be used as a tool to improve HF diagnosis, as lower 6MWT distance may reflect a possible underlying HF diagnosis, as shown by the elevated NT-pro BNP and troponin levels among patients walking less than 345 m, both of which are well-established markers of cardiac dysfunction and heart failure^20^. Elevated troponin (although very mild elevations, as our patients had) may also indicate the possibility of ischemic heart disease, often represent underlying cardiac changes/HF and mismatch between demand/supply (and may not be due to any instable plaque) which should be sought as a cause of exercise intolerance^21^.

The association of the 6MWT distance with higher rates of cardiovascular events is noteworthy, because the 6MWT is an inexpensive and easy to perform test that can provide important prognostic information. This evidences justifies moving forward with prospective and interventional work in the prevention and treatment of cardiovascular risk in patients who have T6MM below 345 m. The 6MWT reflects exercise tolerance, which can be limited by several cardiovascular and non-cardiovascular factors such as cardiac as respiratory diseases, osteoarticular pathology, obesity, physical conditioning, and/or willingness/motivation to perform the test. In addition, the 6MWT also relies on the ability of skeletal muscle to extract oxygen from blood, which may also have prognostic impact^22^.

In the future, 6MWT can be used as a follow-up tool—for the treatment of RA and its comorbidities, such as obesity, anemia or HF, and also to measure the impact of improving outcomes.

In summury. the 6MWT is simple, reproducible, and inexpensive, easily performed in routine practice, and provides important information regarding the patients´ status and outcomes, enabling the monitorization of the therapeutic optimization of the various domains of the RA.

Several limitations should be acknowledged in our study. This is an observational study and the associations with 6MWT were cross-sectional, thus causality cannot be ascertained. Other relevant determinants of exercise capacity, such as skeletal muscle abnormalities and non-physiologic factors, including patient motivation and investigator encouragement could play an important role and were not available. The small number of cases resulting in hard CV outcomes requires caution in interpreting models with many numbers of variables. Our cohort came from a single center and some of the findings may reflect specific socio-demographic characteristics and local practice patterns which may limit the generalization of the findings. Finally, we did not perform the Time Up and Go or Grip Strength tests in our study, which would also be inexpensive and easy to perform, and could have provided important clinical and prognostic information. Further studies should assess the prognostic value of such tests in patients with RA.

## Methods

### Study design

We conducted a prospective single-center cohort study with patients attending the Autoimmune Disease Outpatient Unit of *Centro Hospitalar do Porto*, Portugal. The study was registered in clinicaltrials.gov under the number NCT03960515^20^.

### Patient selection

We considered for inclusion all adult patients (18 years or older) with a diagnosis of RA and that were followed-up in our specialized RA clinic. The RA was diagnosed using the 2010 ACR/EULAR Classification Criteria^23^. All patients presenting an active neoplasm or other severe co-morbid condition potentially associated with a life expectancy shorter than six months, as well as severe dementia, inability to walk or totally dependent on a third person were excluded from our study.

The screening period for the patient enrollment occurred from June 2016 to June 2018. All enrolled patients maintained their usual care under the guidance of their treating physician.

A total of 399 were initially studied, but only patients who underwent 6MWT were included in the study and were followed-up for a median of 1.5 (0.7–2.3) years. The cause of death and hospitalization were independently adjudicated.

### Patient evaluation, echocardiography, and routine laboratory tests

We collected demographic data, cardiovascular history and risk factors, comorbid conditions, RA history, daily medication intake and a cardiovascular symptom questionnaire. Physical examination included vital signs, pulmonary and cardiac auscultation, and anthropometric measures. Transthoracic echocardiogram (TTE) was performed by an experienced echocardiographer and included the M-mode, 2D and Doppler measurements acquired according to standard recommendations^24,25^. Routine laboratory tests included a complete blood count, c-reactive protein, glucose, blood electrolytes, lipid profile, creatinine, high sensitivity troponin T (Elecsys Roche Diagnostics®), and N-Terminal-pro Brain Natriuretic Peptide (NT-pro BNP; Elecsys Roche Diagnostics®).

### 6-Minute walk test

Six-minute walking test was performed according to the American Thoracic Society protocol^26^, in a long, straight hospital corridor, over a 30-m distance. Each participant was asked to walk (not run) back and forth along the corridor as briskly as possible, so that the longest possible distance was covered in 6 min. The participant could slow down or stop and rest if necessary, particularly in the case of symptoms such as severe dyspnea or fatigue. During any rest period, the participant was informed of the elapsed time and encouraged to recommence walking when symptoms subsided sufficiently. The participant could discontinue the test at any time. Moreover, the test was interrupted by the investigator immediately if one of the following symptoms appeared: chest pain, severe dyspnea, severe claudication, loss of balance, severe sweating, pallor, or cyanosis. Otherwise, every two minutes during the test, the investigator informed the participant of the amount of time left and encouraged them to continue the test. At 6 min, the participant was advised to stop and sit down. The distance walked was measured in meters (m) to the nearest whole metre.

### Statistical analysis

Sample description and comparison of the 6MWT according to tertiles was performed using Chi-square and Kruskal–Wallis tests, as appropriate. Continuous variables are presented by median (interquartile range) and categorical by absolute and relative frequency. Single imputation using median was used to deal with missing data. All variables with a P-value of less than 0.05 in the univariable analysis (Table 1) were entered in a stepwise (forward) model with a P-value of less than 0.05 set for a variable to enter and stay in the final model. Using this procedure, we performed a multivariable linear regression model using 6MWT distance as a continuous variable (per each minus 10 m walked) and a logistic regression model (using the lowest tertile distance as outcome of interest). Cox proportional hazard regression models and Kaplan–Meier curves were used to study the association of 6MWT distance with cardiovascular outcomes, a composite of time-to-first cardiovascular death or cardiovascular hospitalization. The covariates used for adjustment where those from the final multivariable models. All the analyses were performed using STATA® (StataCorp. 2019. Stata Statistical Software: Release 16.1. College Station, TX: StataCorp LP). A P-value < 0.05 was considered as statistically significant.

### Ethical approval and consent to participate

This study was conducted following the principles of the Declaration of Helsinki and approved by the *Hospital de Santo António, Porto, Portugal* ethics committee under number #2016-023 (020-DEFI/020-CES). All participants provided the written informed consent before enrollment in the study. The data used for this study are available upon reasonable request to the corresponding author.

## Supplementary Information

Supplementary Table 1.

## Data Availability

The authors state they have full control of all primary data and agree to allow the journal to review their data after reasonable request.

## Abbreviations

6MWT: 6-Minute walk test
RA: Rheumatoid Arthritis
ACR/EULAR: American College of Rheumatology/European League Against Rheumatism
NT-pro BNP: N-Terminal-pro Brain Natriuretic Peptide
TTE: Transthoracic echocardiogram
HF: Heart Failure
ACEi/ARBs: Angiotensin converting enzyme inhibitors/angiotensin receptor blockers
DAS28: Disease Activity Score 28
