# Supplementary material for: Clinical associations and prognostic implications of 6-minute walk test in rheumatoid arthritis
Source: Sci Rep. 2022 Nov 4;12:18672. doi: 10.1038/s41598-022-21547-z (PMC9636394; doi:10.1038/s41598-022-21547-z)
Supplement: Supplementary file 1 — Supplementary Table 1. [file 41598_2022_21547_MOESM1_ESM.docx]

**Supplemental Material**

Supplemental Table 1. Association of 6MWT tertiles with “hard” cardiovascular outcomes

| **6MWT** | **HR (95%CI)** | **P-value** |
| --- | --- | --- |
| >405m | Ref. | - |
| 345-405m | 1.80 (0.53-6.13) | 0.35 |
| <345m | 7.69 (2.69-22.0) | <0.001 |

Legend: 6MWT, 6-minute walking test.

Caption: only a walking distance of less than 345 meters was associated with poor cardiovascular outcomes.

“Hard” cardiovascular outcome is a composite of cardiovascular death or hospitalisation for cardiovascular causes.
